# Supplementary material for: Abnormal Splicing of GALC Transcripts Underlies Unusual Cases of Krabbe Disease
Source: Biomedicines. 2025 Dec 17;13(12):3114. doi: 10.3390/biomedicines13123114 (PMC12731088; doi:10.3390/biomedicines13123114)
Supplement: Supplementary file 1 [file biomedicines-13-03114-s001.zip › Supplementary information v3.pdf]

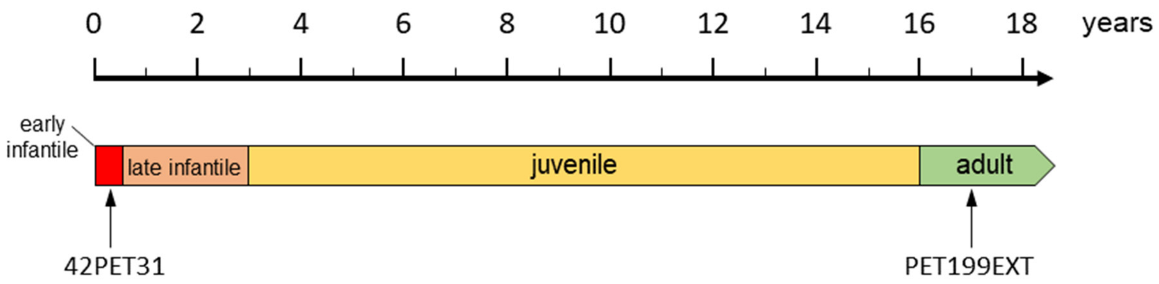

Figure S1. Phenotypes of Krabbe disease across the lifespan

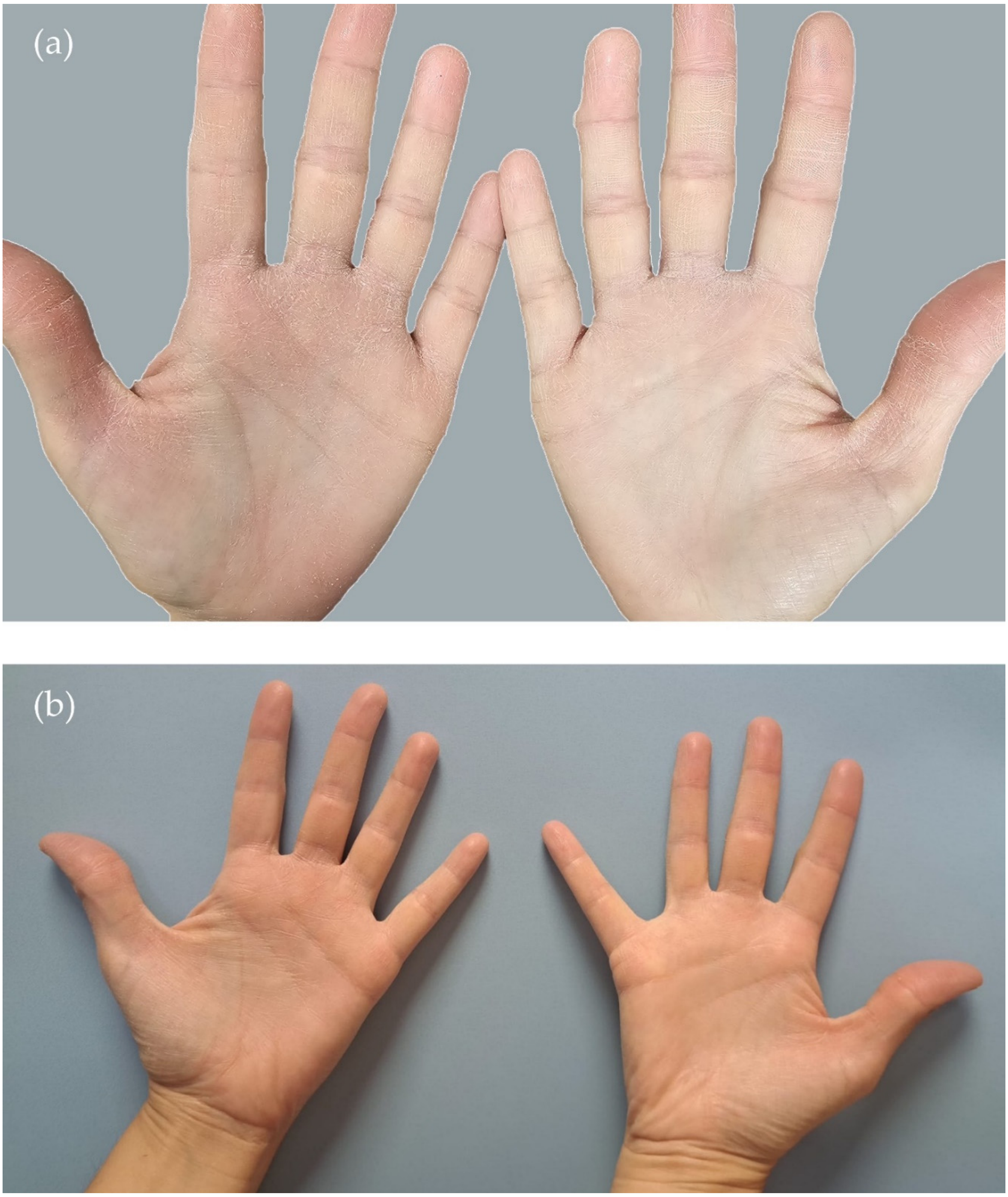

Figure S2. Palms of patient PET199EXT II:1. (**a**) At first consultation with the dermatologist. Note exaggerated skin lines (hyperlinearity). (**b**) After one month of treatment with a ceramide ointment.

Table S1. List of the 101 genes anayzed by the STP v3 panel.

STP Panel v 3

Gene #	gene symbol	Gene #	gene symbol	Gene #	gene symbol
1	*A4GALT*	49	*IDUA*	97	*TPP1*
2	*ABCA1*	50	*ITCH*	98	*UGCG*
3	*ABHD5*	51	*KIT*	99	*UNC13D*
4	*ACER1*	52	*LAMP1*	100	*VDR*
5	*AGA*	53	*LAMP2*	101	*VPS35*
6	*AK1*	54	*LAMP3*		
7	*ANK1*	55	*LIPA*		
8	*APOE*	56	*LRP2*		
9	*AP3B1*	57	*LRRK2*		
10	*ARSA*	58	*LYST*		
11	*ARSB*	59	*MAN2B1*		
12	*ASAH1*	60	*MANBA*		
13	*ASAH2*	61	*MCOLN1*		
14	*BIN1*	62	*MRC1*		
15	*CHIT1*	63	*MTX1*		
16	*CLCN7*	64	*NAGA*		
17	*CLN3*	65	*NAGLU*		
18	*CLN5*	66	*NEU1*		
19	*CLN6*	67	*NPC1*		
20	*CLN8*	68	*NPC2*		
21	*CTNS*	69	*NPR2*		
22	*CTSA*	70	*NT5C3A*		
23	*CTSK*	71	*PARK7 (DJ1)*		
24	*EPB41*	72	*PIEZO1*		
25	*EPB42*	73	*PINK1*		
26	*FUCA1*	74	*PKLR*		
27	*G6PD*	75	*PPT1*		
28	*GAA*	76	*PRF1*		
29	*GALC*	77	*PRKN (PARK2)*		
30	*GALNS*	78	*PSAP*		
31	*GATA1*	79	*RAB27A*		
32	*GBA*	80	*SCARB2*		
33	*GBA2*	81	*SGSH*		
34	*GBA3*	82	*SLC17A5*		
35	*GLA*	83	*SLC4A1*		
36	*GLB1*	84	*SMPD1*		
37	*GM2A*	85	*SNCA*		
38	*GNPTAB*	86	*SNX10*		
39	*GNPTG*	87	*SORT1*		
40	*GNS*	88	*SPTA1*		
41	*GPI*	89	*SPTB*		
42	*GRN*	90	*STX11*		
43	*GUSB*	91	*STXBP2*		
44	*HEXA*	92	*SUMF1*		
45	*HEXB*	93	*TCIRG1*		
46	*HGSNAT*	94	*TFEB*		
47	*HYAL1*	95	*TMEM175*		
48	*IDS*	96	*TNFSF11*		

Lysosomal storage disorders	49
Erythrocyte membrane disorders	6
Hemolytic anemia	6
Other Hematological Disorders	8
Other disorders	8
Involved in lysosomal function	17
Biomarkers	1
Parkinson disease	6
**Total**	**101**

Table S2. Primers and PCR conditions for *GALC* genomic amplicons with causative variants

Target	Primer sequences (5′-3′)	Annealing T (°C)	Amplicon (bp)
Intron 6	Upper: GCAAAGAGGTAGCAGTAACATCT<br>Lower: AGAACATAAAACATCCAATTCAAA	55	857
Exon 8	Upper: GGAAGCTACCCAACTGTTT<br>Lower: TAGATGACGCTAACAAGGC	55	424
Exon 9	Upper: TTTGGGTGATCCTTTTATTGTCATT<br>Lower: TCCCCCTCCTCTCCTACACTT T	55	482
Exon 10	Upper: GGGTTGGGACATGGTATATTGC<br>Lower: GAGGTTCCCTAAGCCATCAGTC	55	585

Table S3. Primers and PCR conditions for overlapping *GALC* cDNA amplicons

Target	Primer sequences (5′-3′)	Annealing T (°C)	Amplicon (bp)
Exons 1 to 5	Upper: CTGAGTGGCTACTCTCGGCTT<br>Lower: CCACAATCCAGGTCACGACA	62	537
Exons 4 to 9	Upper: TATTTCCGAGGATACGAGTGGT<br>Lower: CATCTCCCATAAGGCAACTGTT	57	593
Exons 8 to 13	Upper: GGGGTCGCATTTTAAATCAGAA<br>Lower: TTTGGAGGAAGCGGGTAGC	57	583
Exons 12 to 15	Upper: CAAACTTGGAAAAACATCCG<br>Lower: ATTCCTCTGGCACTTCTAATCA	57	501 (full-length) or<br>320 (minus exon 14)
Exons 14 to 17	Upper: CGCCAAGTTCTCAACCAG<br>Lower: AAGCCTCATATACTGTTCCAATG	57	568
Exons 7 to 9	Upper: TTATCAAGGTCTCCAGCGAGTG<br>Lower: CCCGCATCTCCCATAAGG	57	328
Exon 5 to 10	Upper: ACTGGCCTTATGTCAATCTT<br>Lower: GCCAACTGTCTTCAGGTAAT	57	602

Table S4. Nested PCR primers for *GBA1* exon 9, annealing temperature 62°C

Upper sequence (5′-3′)	Lower sequence (5′-3′)
CTTCCTGCTCCCTCGTGGTGTA	TGACCTACCCACAGCTGCCTCT

Table S5. Description of ACMG criteria used in this article [25]

Criterion	Strength	Definition
PVS1	Very strong	Null variant (nonsense, frameshift, canonical ±1 or 2 splice sites, initiation codon, single or multiexon deletion) in a gene where loss of function is a known mechanism of disease
PS3	Strong	Well-established in vitro or in vivo functional studies supportive of a damaging effect on the gene or gene product
PM1	Moderate	Located in a mutational hot spot and/or critical and well-established functional domain (e.g., active site of an enzyme) without benign variation
PM2	Moderate	Absent from controls (or at extremely low frequency if recessive) in Exome Sequencing Project, 1000 Genomes Project, or Exome Aggregation Consortium
PM3	Moderate	For recessive disorders, detected in *trans* with a pathogenic variant
PM4	Moderate	Protein length changes as a result of in-frame deletions/insertions in a nonrepeat region or stop-loss variants
PP3	Supporting	Multiple lines of computational evidence support a deleterious effect on the gene or gene product
PP4	Supporting	Patient’s phenotype or family history is highly specific for a disease with a single genetic etiology
PP5	Supporting	Reputable source recently reports variant as pathogenic, but the evidence is not available to the laboratory to perform an independent evaluation

Table S6. Genotype-phenotype correlations in previous reports of variant c.956A>G in *GALC*

Allele 1	Allele 2	Age of onset	Phenotype	Initial symptoms	Reference
c.956A>G	c.956A>G	~2½ years	Late infantile	Visual impairment	[45]
c.956A>G	c.956A>G	~5½ years	Juvenile	Visual impairment	[45]
c.956A>G	c.956A>G	~4 years	Juvenile	Visual impairment	[45]
c.956A>G	30-kb del[1]	Not reported	Infantile (?)	Not reported	[44]
c.956A>G	30-kb del	~5 years	Juvenile	Visual impairment	[49]
c.956A>G	30-kb del	~5 years	Juvenile	Visual impairment	[49]
c.956A>G	30-kb del	~12 years	Juvenile	Spastic paresis	[49]
c.956A>G	30-kb del	~23 years	Adult	Walking difficulty	[50]
c.956A>G	30-kb del	~23 years	Adult	Walking difficulty	[50]
c.956A>G	c.860+1G>A	29 months	Late infantile	Regression of milestones	[51]
c.956A>G	c.296+1G>T	~12 years	Juvenile	Visual impairment	[45]
c.956A>G	c.1230del	14 months	Late infantile	Regression of milestones	[46]
c.956A>G	c.626T>A	~6 years	Juvenile	Walking difficulty	[46]
c.956A>G	c.1034-1G>C	~17 years	Adult	Walking difficulty	This work

[1] 30-kb del: c.1161+6532_polyA+9kbdel
